# Cardiovascular Programming During and After Diabetic Pregnancy: Role of Placental Dysfunction and IUGR

**DOI:** 10.3389/fendo.2019.00215

**Published:** 2019-04-09

**Authors:** Immaculate M. Langmia, Kristin Kräker, Sara E. Weiss, Nadine Haase, Till Schütte, Florian Herse, Ralf Dechend

**Affiliations:** ^1^Experimental and Clinical Research Center, A Joint Cooperation Between the Max-Delbrueck Center for Molecular Medicine and the Charité Universitätsmedizin Berlin, Berlin, Germany; ^2^Berlin Institute of Health (BIH), Berlin, Germany; ^3^Alexander von Humboldt Foundation, Bonn, Germany; ^4^Charité–Universitätsmedizin Berlin, Corporate member of Freie Universität Berlin, Berlin Institute of Health, Humboldt-Universität zu Berlin, Berlin, Germany; ^5^Max-Delbrueck Center for Molecular Medicine in the Helmholtz Association, Berlin, Germany; ^6^DZHK (German Centre for Cardiovascular Research), Partner Site Berlin, Berlin, Germany; ^7^Center for Cardiovascular Research, Institute of Pharmacology, Charité -Universitätsmedizin Berlin, Berlin, Germany; ^8^HELIOS-Klinikum, Berlin, Germany

**Keywords:** diabetes, placental dysfunction, gene modification, intra uterine growth restriction, cardiovascular programming

## Abstract

Intrauterine growth restriction (IUGR) is a condition whereby a fetus is unable to achieve its genetically determined potential size. IUGR is a global health challenge due to high mortality and morbidity amongst affected neonates. It is a multifactorial condition caused by maternal, fetal, placental, and genetic confounders. Babies born of diabetic pregnancies are usually large for gestational age but under certain conditions whereby prolonged uncontrolled hyperglycemia leads to placental dysfunction, the outcome of the pregnancy is an intrauterine growth restricted fetus with clinical features of malnutrition. Placental dysfunction leads to undernutrition and hypoxia, which triggers gene modification in the developing fetus due to fetal adaptation to adverse *utero* environmental conditions. Thus, *in utero* gene modification results in future cardiovascular programming in postnatal and adult life. Ongoing research aims to broaden our understanding of the molecular mechanisms and pathological pathways involved in fetal programming due to IUGR. There is a need for the development of effective preventive and therapeutic strategies for the management of growth-restricted infants. Information on the mechanisms involved with *in utero* epigenetic modification leading to development of cardiovascular disease in adult life will increase our understanding and allow the identification of susceptible individuals as well as the design of targeted prevention strategies. This article aims to systematically review the latest molecular mechanisms involved in the pathogenesis of IUGR in cardiovascular programming. Animal models of IUGR that used nutrient restriction and hypoxia to mimic the clinical conditions in humans of reduced flow of nutrients and oxygen to the fetus will be discussed in terms of cardiac remodeling and epigenetic programming of cardiovascular disease. Experimental evidence of long-term fetal programming due to IUGR will also be included.

## Fetal Cardiovascular Programming in Conditions of Placental Dysfunction and IUGR

Cardiovascular disease (CVD) is the main cause of mortality and morbidity in the twenty-first century (1–3). According to the World Health Organization (WHO), coronary heart disease and stroke accounted for ~15.2 million deaths in 2016 globally. Previously, CVD was assumed to be caused by a series of events that occur after birth, such as a person's lifestyle, age, and other disease conditions. In other words, postnatal gene-environment interactions are important factors promoting CVD, but recently we began to understand that *in utero* environmental changes that occur in the first few months of prenatal development epigenetically program the fetus for development of CVD in adulthood (4–6). Therefore, apart from postnatal gene-environment interaction, information on *in utero* gene-environment interactions is needed to improve our understanding of CVD.

The placenta performs many functions ranging from growth of the fetus, prevention of fetal rejection by maternal immune system, as well as the transport/exchange of gases, nutrients, and waste products between mother and fetus (7). In addition, the placenta is involved in metabolism and production of many hormones that play vital roles in the maintenance of pregnancy (7). In a normal pregnancy the placental weight and birth weight are highly correlated (8). In adverse situations where the maternal-fetal circulation is altered due to disease conditions such as diabetes, the development of the placenta also changes (9). The determining factor in placental dysfunction is the extent of exposure to hyperglycemia during fetal and placental development (10). Depending on the degree of hyperglycemia, the growth of the placenta and the fetus can be severely affected (10). Long-term uncontrolled hyperglycemia can lead to placental vascular dysfunction in women suffering from diabetes (10). Endothelial dysfunction was observed in hyperglycemic human umbilical vein cells (HUVEC) compared to control cells (11). According to studies by Starikov et al. pre-gestational diabetes led to placentas that were small for gestational age in 20% of pregnancies and placentas that were large for gestational age in 30% of cases (9). In conditions where maternal hyperglycemia is not controlled, it results in histological changes of the placental tissues (10, 12). Placentas from mothers with diabetes display different histological features such as immature villous, increased number of fetal capillaries, and fibrinoid necrosis of the placental villi (7). Prolonged maternal insults such as hyperglycemia, dyslipidemia, and hyperinsulinemia may exceed the placental capacity to adapt and respond leading to placental dysfunction and adverse fetal outcome such as in IUGR (13, 14). Placental dysfunction is the most common cause of asymmetrical intrauterine growth restriction (15–17). Uteroplacental malperfusion, decidual vasculopathy, placental infarct, and maternal vasculopathy were observed in women with diabetes (12) and are associated with reduced fetal growth and adverse outcome (17, 18). Abnormal placental vascular development decreases normal placental blood supply, leading to reduced oxygen (hypoxia) and nutrient delivery (undernutrition) to the fetus and a subsequent IUGR fetus (16, 17). As a result of reduced oxygen and nutrition, the fetus redistributes its cardiac output to increase oxygen and nutrient supply to the brain, which is referred to as brain sparing (19). This prenatal cerebral circulatory adaptation has been associated with long-term behavioral problems in schoolchildren who were growth-restricted at birth. These difficulties include problems in memory, cognition, motor skills, and neuropsychological malfunction (20). IUGR offspring experience the highest rate of coronary heart disease, myocardial dysfunction, type 2 diabetes, hypertension, and stroke as adults (21, 22). The underlying molecular mechanisms leading to fetal susceptibility to adult disease are still under investigation. Interestingly, we now know that clinical symptoms of cardiovascular disease may not appear until adult life (4). In 1989, David Barker demonstrated this aspect amongst individuals with low birth weight, showing a direct association between low birth weight and CVD in adulthood. This study demonstrated that the incidence of ischemic heart disease and death were three times higher among men with low birth weight compared to men with high birth weight (5). Epidemiological investigations of adults born at the time of the Dutch famine between 1944 and 1945 revealed an association between maternal starvation and a low infant birth weight with a high incidence of hypertension and coronary heart disease in these adults (23). Furthermore, Painter et al. reported the incidence of early onset coronary heart disease among persons conceived during the Dutch famine (24). In that regard, Barker's findings led to the concept of fetal adaptation to prenatal environmental changes and vulnerability to chronic diseases in adult life, a concept known as “fetal programming.” In 2003, the concept of “Developmental origins of health and disease (DOHaD)” was published (25). DOHaD describes the scenario whereby *in utero* maternal insults result in structural and functional changes in fetal organs extending in postnatal life and increasing susceptibility of chronic disease in adulthood. Fetal programming of other organs such as the brain (19), lungs (26), and hypothalamic-pituitary-adrenal (HPA) axis have been greatly studied, however the heart is also very important and few studies have investigated fetal programming of the heart as summarized below (27).

## Fetal Programming of the Heart in IUGR

The heart is the first organ to develop and function during embryogenesis (28). Fetal organs that develop early are more vulnerable to changes of the *in utero* environment than organs that develop later (29). There is a direct correlation between the amount of oxygen and nutrient supply to the fetus and cardiomyocytes development and function (29). Chronic hyperglycemia causes placental vascular dysfunction, which leads to reduced nutrient and oxygen supply to the fetus resulting in IUGR (10). Neonates exposed to IUGR present significant changes in cardiac morphology and subclinical myocardial dysfunction at birth (29). As such, in placental dysfunction due to maternal diabetes involving undernutrition and hypoxia, the developing myocardium undergoes structural and functional changes referred to as cardiac remodeling (30). In 1982, Hochman and Bulkley were the first to use the term “remodeling” to describe the substitution of infarcted tissue with scar tissue in a model of myocardial infarction (31). Later, in the year 2000, an international forum defined cardiac remodeling as molecular, cellular, and interstitial changes that manifest clinically as changes in size, shape, and function of the heart resulting from cardiac load and injury (30). Therefore, the clinical phenotype is a consequence of genomic changes that occur due to *in utero* gene environment interaction. Few clinical studies and experimental animal models have investigated the impact of IUGR on cardiac remodeling as discussed below.

## Impact of Placental Dysfunction on Cardiac Structure and Function

Epidemiological studies have shown changes in the structure and function of the heart in IUGR infants and neonates (29, 32). Clinical investigations have reported that postnatal catch-up growth in IUGR infants can result in abnormal cardiac function (33–35). Cardiovascular evaluation using echocardiographic assessment of IUGR fetuses revealed cardiac hypertrophy characterized by globular cardiac shape, higher intraventricular septum thickness, and increased pressure overload leading to both systolic and diastolic dysfunction (36–38). Primary cardiac and vascular changes observed at birth in IUGR fetus can persist from a few months up to a few years of age (35, 37).

Recently, clinical investigations have shown evidence of cardiac remodeling in IUGR offspring (32). For example, high systolic blood pressure and smaller aortic diameter (ascending aorta; IUGR:24.4 ± 1.5 mm, Control: 26.3 ± 2.2 mm, *P* < 0.05) were observed 20 years later in young adults (age 22–25 years) who were born growth restricted due to placental dysfunction (32). Compared to healthy controls, the diameter of the ascending aorta was smaller in IUGR subjects and a high aortic pressure augmentation index was observed (32). Electrocardiography (ECG) measurement revealed electrical remodeling in preadolescence who were born with IUGR (39). These studies evaluated signs of ventricular electrical remodeling in subjects with IUGR compared to controls by assessing changes in ventricular depolarization and repolarization using the QRS complex and *T*-wave, respectively (39). The classical frontal QRS-T angle was significantly narrower in IUGR preadolescents compared to control subjects (39). They also reported wider angles between the depolarization dominant vector and the frontal XY body plane in preterm-IUGR subjects, resulting in significantly wider angles between depolarization and repolarization vectors (39). The studies concluded that ventricular electrical remodeling observed in the pre-adolescent subjects might predispose to cardiovascular disease in later life (39). Electrical remodeling is induced by functional (altered electrical activation) and structural stressors (such as in hypertrophy and heart failure). These electrophysiological changes generate a substrate that is vulnerable to malignant ventricular arrhythmias. Cardiac remodeling and dysfunction increases with greater severity of IUGR (33, 40). The above-mentioned studies show evidence of cardiac remodeling in neonates, pre-adolescents, and young adults who were born with IUGR (41–44). These findings are supported by animal models; IUGR programming resulted in myocardial remodeling, reduced systolic and diastolic function and premature aging of the heart in growth restricted baboons (45). These structural and functional changes of the heart can be associated to changes in fetal cardiac gene expression that have occurred because of fetal adaptations to intrauterine environmental changes (46). These *in utero* changes, which result in reduced nutrition and oxygen, are due to placental dysfunction caused by maternal insults such as maternal vasculopathy in a diabetic pregnancy (46).

## Differential Expression of Cardiac Genes Leading to Dysfunctional Heart in IUGR

Pre-gestational and gestational diabetes have been associated with maternal vasculopathy which can be caused by long-term poor glycemic control (12). Fetal growth in diabetic pregnancy might be affected in two different ways; maternal hyperglycemia stimulates fetal overgrowth and maternal vasculopathy may be associated with placental dysfunction leading to a reduced nutrient and oxygen supply and subsequent IUGR (12). The majority of animal models use either maternal undernutrition and/or hypoxia to mimic IUGR (6, 47, 48). Hypoxia, undernutrition, or both interventions induce cardiac remodeling in adult rats (47). IUGR models have linked cardiac remodeling with changes in fetal gene expression. Several genes that play key roles in cardiac development have been studied in detail. One prominent example is the hypoxia-inducible factor 1 (HIF-1), which is required for normal growth of the myocardium and coronary blood vessels in conditions of low oxygen (49, 50). High levels of HIF-1 were reported in fetal rodent hearts exposed to hypoxia (49, 50). Abnormal cardiogenesis was seen in HIF-1 alpha-deficient mice (51). Therefore, elevated expression of *HIF1* and its downstream genes is essential for fetal adaptation and proper cardiac development in conditions of placental dysfunction in which oxygen supply to the fetus is relatively low (51–55). In a guinea pig model of maternal nutrient restriction, hearts of growth restricted offspring revealed increased expression of Poly [ADP-ribose] polymerase 1 (*PARP1)* gene and a reduction in cardiomyocyte number as well as hypertrophy (56). Another interesting class of genes associated with cardiac remodeling are the cardioprotective genes such as heat shock protein 70 (*HSP70*) and protein kinase C epsilon (*PKC*ε) (57). Male rats exposed to hypoxia had increased expression of HSP70 and ischemic injury (48, 58–60). In addition, hypoxia in rat hearts resulted in a significant increase in *PKC*ε expression and increased susceptibility to ischemia and perfusion injury in a sex-dependent manner (61). Furthermore, the mammalian target of the rapamycin complex 1 (mTORC1) pathway has also been associated with cardiac remodeling in IUGR mice, and prenatal *mTORC1* inhibition caused a reduction in cardiomyocyte number in adult hearts compared to controls (62). In a rabbit model, IUGR was associated with cardiac mitochondrial Complex II dysfunction and an increase in Sirtuin 3 expression (63). In a rat model, maternal diabetes was associated with increased expression of forkhead box protein O1 (*FOXO1)* and its target genes in the heart of the offspring (64). Under conditions of undernutrition and hypoxia, changes that occur to enable the growing fetus to adapt and survive the inadequate substrate supply results in programming of fetal cardiac genes (4, 6, 65–67).

## Role of Epigenetics in Cardiac Programming

Researchers have been investigating the mechanisms by which IUGR caused changes in fetal cardiac genes. Epigenetic regulation is one principle underlying molecular mechanism that causes differential gene expression in IUGR fetuses compared to normally grown fetuses. Epigenetic activities can alter gene expression throughout an individual's lifetime; thus, the function of the corresponding protein products as well as the organs involved are affected. Gene modifications occur in response to environmental changes, and genes are switched on and off via epigenetic mechanisms. Epigenetic alterations could be altered by lifestyle (for example overnutrition or undernutrition) or possibly by using treatments strategies postnatally (68). Hence, it is proposed that epigenetic mechanisms are important for understanding pathophysiology as well as potential targets for diagnosis and treatment of cardiovascular diseases (69, 70). Epigenetic regulation occurs by DNA methylation, histone modification, or regulation of genes by non-coding RNAs (ncRNAs) such as small microRNA and long non-coding RNA (71). So far, DNA methylation and histone acetylation are the most studied epigenetic mechanisms and clinical evidence of epigenetic programming is reported in adult patients with various cardiovascular diseases as reviewed by Muka et al. (72). Other studies have investigated the posttranscriptional regulatory role of non-coding RNA in relation to cardiovascular disease susceptibility in adult cardiac patients (73–75). We will focus on DNA methylation in IUGR leading to cardiovascular disease.

## DNA Methylation Mechanisms in IUGR Leading to Development of Cardiovascular Disease

**Figure 1:**
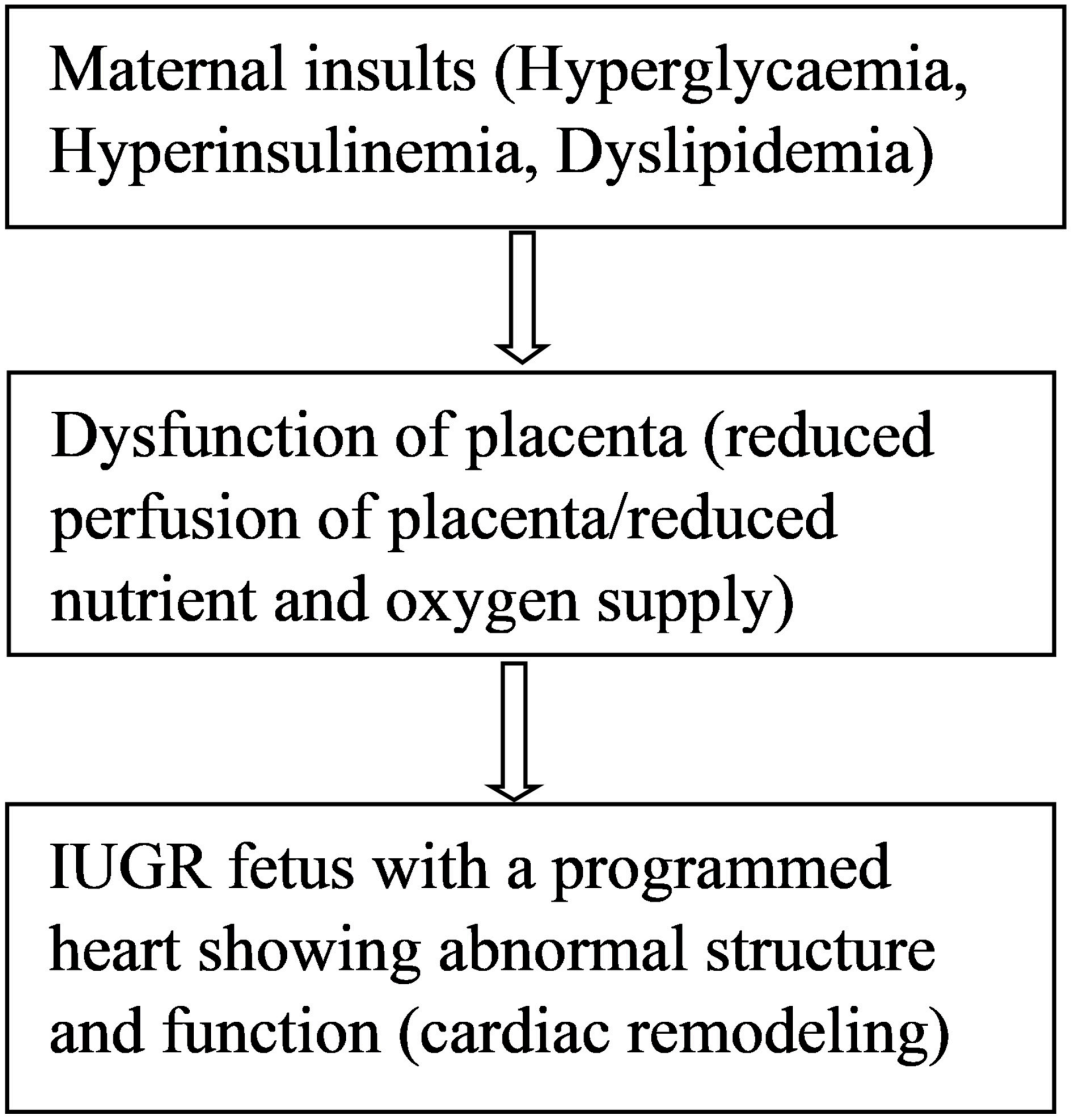
Maternal insults result in placental dysfunction, IUGR, and cardiac remodeling.

DNA methylation is the covalent addition of methyl groups to the C5 position of cytosine in dinucleotide CpG islands (76). CpG islands are regions with a high frequency of CpG sites where a cytosine nucleotide is followed by a guanine nucleotide. Methylation of CpG sites is catalyzed by DNA methyl transferases (DNMTs), such as DNMT1, DNMT3a, and DNMT3b. Very few studies have examined DNA methylation mechanisms in IUGR leading to cardiovascular remodeling or cardiac dysfunction. In a UK cohort of 144 children, investigators used umbilical cord blood to examine the relationship between prenatal antisense non-coding RNA in the INK4 locus (*ANRIL)* promoter DNA methylation and risk markers of coronary heart disease (76). Hypermethylation at CpG5 of the *ANRIL* promoter was associated with increased childhood pulse wave velocity, indicating increased arterial stiffness and a risk of coronary heart disease in these children at 9 years old (76). The *ANRIL* promoter is present on chromosome 2p21 which is considered a strong candidate for coronary heart disease in adult patients (77). Methylation of *ANRIL* and other genes on this chromosome might contribute to the prolonged cardiovascular programming in coronary disease patients. However, it is difficult to conclude the epigenetic contribution unless studies involving long-term follow-up of IUGR individuals are performed. Several studies have investigated epigenetic disturbances of nitric oxide synthase (NOS), which may predispose individuals to cardiovascular disease by modulating endothelial dysfunction (78–80). Human endothelial cells isolated from umbilical arteries and veins of IUGR fetuses were analyzed for a DNA methylation pattern in the promoter region of the endothelial nitric oxide synthase 3 (*eNOS3*) and arginine-2 (*ARG2*) genes. Compared to control cells, differential DNA methylation at the CpG-352 site of *NOS3* gene promoter of IUGR-endothelial cells was observed (80). Differential *eNOS* expression could be normalized by simply silencing the DNA methylation machinery (80). Hypoxia downregulates *eNOS* gene expression and activity, resulting in a reduced production of nitric oxide and endothelial dysfunction (78). Endothelial dysfunction is a very early stage in the development of atherosclerosis, which appears prior to the existence of atherosclerotic plaques or cardiovascular outcomes (81–84). This favors the concept that an epigenetic marker detectable as early as during prenatal and early postnatal developmental periods might be capable of identifying persons at risk of endothelial-related cardiovascular end organ damage. Furthermore, it has been shown that hypoxia-related IUGR changes the methylation pattern and expression of the *PKC*ε gene in rat hearts, causing ischemic injuries in offspring (85). Hypoxia causes increased production of reactive oxygen species (ROS) which induces epigenetic repression of the PKCε gene leading to susceptibility of the heart to ischemic injury in the offspring (85). Hypermethylation patterns were observed within the promoter region of the *PKC*ε gene in IUGR male rat hearts compared to controls (85). *PKC*ε promoter hypermethylation was associated with a corresponding down regulation of *PKC*ε gene expression in the heart of male rats but not in females. Activation of the *PKC*ε gene in the heart restored hypoxia-induced cardiac vulnerability to ischemic injury in male rats (85). The transcription factor, early growth response factor (Egr-1), is involved in the regulation of *PKCe* promoter activity (86) since it binds to the *PKC*ε promoter region, increasing its activity. Further studies showed that the absence of methylation in females was due to high levels of estrogen receptors in the female rat heart. Estrogen receptors bind to the regulatory gene Egr-1 and inhibit *PKC*ε promoter methylation activity (86). Therefore, compared to males, the female hearts are protected against hypoxia-induced ischemic injury due to high levels of estrogen. These data help to explain the differences in the prevalence of CVD between men and women and gives a molecular mechanism for the role of sex hormone (87). Postmenopausal women seem to lose this hormonally-induced cardioprotective ability and as such, hormone therapy is suggested as one of the remedies for prevention of cardiovascular disease in this group of women (88). However, large studies do not favor hormone replacement therapy for prevention of cardiovascular disease since it also has side effects such as increased risk for breast cancer and pulmonary embolism (88). For IUGR epigenetically programmed hearts, the best preventive strategy would be the use of epigenetic therapies (drugs) that aim to reverse fetal heart programming in early postnatal life (89). Postnatal drugs are capable of altering the epigenetic modifications that occurred during prenatal life as a result of fetal adaptations to changes in the intrauterine environment (89). Animal models and human studies of IUGR revealed epigenetic changes in 10 genes which result in a predisposition to type 2 diabetes as reviewed in detail by Liguori et al. (90). These epigenetic mechanisms may further contribute to the high prevalence of cardiovascular disease in type 2 diabetes patients (90). Another gene involved in growth and development of the fetal heart is insulin-like growth factor *(IGF*). Upregulation of cardiac IGF receptor is associated with an increase in ventricular mass in a sheep model of nutrient restriction (91). Moreover, epigenetic mechanisms in the renin angiotensin system are linked to cardiovascular programming. Animal models revealed epigenetic modification of the renin-angiotensin system in fetal programming of hypertension (92, 93). Significantly lower renal expression of angiotensin II type 2 receptor, impairment of renal development and elevation of blood pressure were observed (93). Maternal insults during pregnancy induce placental dysfunction, leading to IUGR (Figure 1) (10). So far, it is well established that IUGR leads to cardiac remodeling via epigenetic modulation of cardiac genes which play important roles in cardiac development and function. However, many epigenetic pathways are still to be elucidated.

## Discussion and Future Perspective

Placental dysfunction due to maternal insults can result in IUGR, which result in an altered fetal epigenome leading to cardiac remodeling and subclinical symptoms of cardiovascular phenotypes in offspring. It is well-proven that IUGR leads to cardiac remodeling, likewise evidence linking altered genome expression and cardiovascular disease in IUGR has been published (85, 86, 91, 94). However, research involving epigenetic mechanisms of cardiovascular programming in growth restricted fetuses has just commenced. To date, most research has focused on fetal programming leading to CVD-related risk factors such as a predisposition to metabolic syndrome (95–97). Although such studies improve our understanding of how to prevent cardiovascular disease in carriers of cardiovascular risk factors, it is important to fully understand the epigenetic mechanisms acting directly on cardiovascular structure and function in IUGR offspring. It seems IUGR results in DNA methylation in a tissue-specific manner; thus, understanding the epigenetic mechanisms in various tissues that have direct impact on the cardiac system will be beneficial (98). The greatest task is to uncover the specific pathways of prenatal methylation of gene expression, as it is vital to the development and function of heart tissues, and to be able to link these pathways to specific cardiac diseases. Even though one can find DNA methylation in interesting CpG island in clinical samples obtained from affected IUGR offspring, it is also difficult to conclude a causal link between these epigenetic modifications and the various heart diseases. However, animal models may help to improve our understanding. Another challenge is linking affected pathways based on the severity of the IUGR since the extent of cardiac remodeling is based on the severity of IUGR (40). Epidemiological time point experiments have been performed to widen our understanding on the role of IUGR in fetal vulnerability to cardiovascular disease (33–35); however, there is need for longitudinal studies involving long-term follow-up of affected IUGR offspring. The use of epigenetic therapies in treatment of CVD is promising but it is still at its early experimental stage. In other diseases such as cancer, systemic lupus erythematosus, acute myeloid leukemia, and Alzheimer's disease, many epigenetic-related therapeutic agents are being tested for their possible use in clinical practice, but many are still awaiting approval to begin clinical trials or to receive approval by the FDA (99–102). In terms of DNA methylation mechanisms, therapeutic agents such as inhibitors of DNA methyl transferase (DNMT), hormonal therapy, and certain dietary compounds have been suggested for treatment of cardiovascular disease (89). Early prediction of cardiovascular disease might reduce risk as well as improve follow-up and treatment of susceptible patients. Effective biomarkers of risk are needed for development of CVD prevention strategies (103). Epigenetic biomarkers could be useful for early prediction of high-risk individuals (103).

Growth restricted newborns are at high risk of respiratory distress syndrome, retinopathy of prematurity, and long-term complications such as metabolic syndrome and cardiovascular disease (104, 105). Therefore, it is vital to identify and follow those who are likely to be affected. Studies also show that an inheritable altered fetal cardiac genome can be transferred from one generation to another, which is referred to as transgenerational programing of the cardiac system (106). “Healthy aging starts with a healthy pregnancy,” summarizes very beautifully this important concept and was first introduced in an editorial of Scioscia (107). A timely diagnosis of IUGR pregnancies and application of various therapeutic measures to treat and monitor affected neonates will help to reduce cardiac problems in future generations.

## Author Contributions

RD and IL conceived the presented idea. IL wrote the first draft of the manuscript. KK, SW, NH, TS, FH, and RD contributed to the manuscript revision, read and approved the submitted version.

### Conflict of Interest Statement

The authors declare that the research was conducted in the absence of any commercial or financial relationships that could be construed as a potential conflict of interest.
